# What is social constructionism about race? A reply to Hochman

**DOI:** 10.1007/s10539-025-09995-z

**Published:** 2025-09-10

**Authors:** Celso Neto

**Affiliations:** 1 Egenis–Centre for the Study of the Life Sciences, University of Exeter, Exeter, UK; 2 Department of Social and Political Sciences, Philosophy and Anthropology, University of Exeter, Exeter, UK

**Keywords:** Race, Social construction, Anti-Realism, Scientific concepts, Investigative practice

## Abstract

This paper reconceptualizes social constructionism about race (hereafter SCR). While SCR is considered a hegemonic view in philosophy and academia more broadly, Hochman (2022) argues that this hegemony is illusory. He identifies different versions of SCR in the literature, showing that race constructionists do not share a single, common view. For him, race constructionists are not even united in rejecting biological race realism, and the label “social constructionism about race” is so inclusive that it has become almost useless. I identify what is missing in Hochman’s analysis, namely, the recognition that SCR is an *investigative practice* (Brigandt 2012; Brigandt and Love 2012; Neto 2020). This recognition demands shifting focus from what race constructionists claim to what they do. By operating this shift, I explain why SCR remains an alternative to biological race realism in a important and specific sense, and why the label “social constructionism about race” is still useful.

## Introduction

Social constructionism about race (hereafter SCR) is a widespread view among natural and social scientists (Mallon 2007; Hochman 2022). Typically, this view treats race and racial groups as products of social factors such as human institutions, norms, and socio-historical processes (Mills 1998; Sundstrom 2002; Haslanger 2000, 2012; Jeffers 2013; Diaz-Leon 2015; Asta 2018). Slavery and colonialism are only two examples of such social factors. Philosophers of race contrast SCR with two other views, namely biological race realism and race anti-realism (Andreasen 2000; Glasgow et al. 2019; Kalewold 2024). Biological race realism treats racial groups as biological divisions in nature, but it finds little support in academia, while race anti-realism proposes that nothing in the world can be legitimately called “race” (Hardimon 2017; Spencer 2018, 2019; Appiah 1985, 1996; Zack 1993; Glasgow 2010).

Adam Hochman is one of the most prolific race anti-realists in philosophy today (2017; 2021; 2022). Hochman offers valuable contributions to the metaphysics debate and proposes “anti-realism reconstructionism,” the idea that races do not exist, but racialized groups do (2017; 2019). This proposal attempts to overcome traditional problems of race anti-realism and incorporate insights from SCR. Hochman also contributes to the metaphysics of race debate by offering extensive criticisms of realist positions (2013; 2014; 2017; 2021; 2022; 2024). His criticisms were initially targeted at new versions of biological realism, but now he turns his attention increasingly against race constructionists (2017; 2021, 2022; 2024). These criticisms are especially interesting given that SCR is a hegemonic view among academics.

In a recent paper, Hochman argues that this hegemony is illusory (2022, 2). He claims that scholars use the expression “social constructionism about race” to denote widely different and even contradictory views about race, such that there is no substantive unifying position among race constructionists. According to Hochman, constructionists are not even united in *rejecting* biological race realism. The ambiguity of “social constructionism about race” is evidence of that disunity, and it leads him to conclude that “any practical usefulness this label may have had has been outlived” (2022, 2).

In this paper, I challenge Hochman’s conclusion. I show that the ambiguity of “social constructionism about race” does not entail a lack of practical usefulness. This expression has an important function, i.e., it identifies a common *investigative practice* shared by many self-declared race constructionists. As I will show, this investigative practice examines the social causes and effects of racialization as a contingent, interactive, and historical process. Moreover, that investigative practice involves explicit normative commitments, and it is a historical reaction to racialism and bio-centric approaches to race in the 20th Century. In this sense, I suggest that SCR can be usefully understood as a shared investigative practice.

By describing this practice, I expand the philosophical treatment of SCR. While philosophers of race tend to emphasize the metaphysical claims and commitments of SCR, I focus on what many self-declared race constructionists do in practice, i.e., *what* they are trying to achieve (e.g., explain) and, most importantly, *how*. I agree with Hochman that race constructionists might adopt different (and even contradictory) views about race. Those scholars only share a minimal metaphysical commitment, as I will explain later. Nevertheless, it is important to recognize that many race constructionists conduct a similar research practice. Recognizing this similarity contributes to a more nuanced analysis of the metaphysics of race debate (Mallon 2022). Furthermore, I show that “social constructionism about race” is an ambiguous but also useful label for highlighting and drawing attention to the shared investigative practice among race constructionists.

This paper proceeds as follows. First, I explain Hochman’s views on SCR and the ambiguity of “social constructionism about race.” Second, I discuss the relationship between ambiguity and the practical usefulness of labels based on recent work on scientific concepts (Brigandt and Love 2012; Feest and Steele 2012; Neto 2020; Novick and Doolittle 2021; Novick 2023). This discussion will offer some examples of useful but highly ambiguous labels. Third, I characterize SCR as a shared investigative practice. This characterization suggests that “social constructionism about race” is even more ambiguous than Hochman thought, but it also indicates why that label is useful. Fourth, I consider some objections and implications of my proposal. For instance, I show that *in a certain specific (and important) sense*, SCR still counts as an alternative to biological race realism. Conceiving SCR as an investigative practice is not meant to offer a single, best definition for race constructionism. Yet, this conception sheds how decisions about research goals and other metatheoretical elements influence the metaphysics of race debate (Mallon 2006, 2022; Ludwig 2015, 2016; Neto 2025).

## Hochman and the ambiguity problem for SCR

The claim that races exist as social constructs seems to be the consensus view in academia, but Hochman thinks this consensus is not genuine (2022, 5). According to him, too many different views can be legitimately described as “social constructionism of race,” making this label too inclusive and almost meaningless. While there might still be something in common among race constructionists, this commonality is not sufficient to identify a single, coherent view and single out those who are not race constructionists.

Hochman’s argument starts with linguistic analysis. He surveys various meanings associated with the expression “social constructionism of race” in the philosophical and scientific literature, identifying four main usages (2022, 4–5):

(i) A view about social influences on racial classification.
(ii) An anti-realist position about biological race.
(iii) A realist view about biological race.
(iv) A realist view about race as a social category.

According to Hochman, (i) refers to a common version of SRC among social and natural scientists (Reich 2018; Carl 2019). This version states that *ideas* about race (e.g., definitions, meanings, classificatory schemes) are man-made and can vary across social contexts (2022, 4). For example, Brazil and the United States have different definitions of “Black,” and these definitions are influenced by social-political factors specific to each country (e.g., the adoption of the so-called “one-drop rule” in the US). This version of SCR is so widely accepted that it would be hard to find someone who is not a race constructionist in this sense (Hochman 2022, p. 9).

The other versions of SCR concern metaphysical views about the existence of race and racial groups as entities in the world. According to version (ii), sometimes the expression “social constructionism about race” conveys the view that races do not exist as biological entities. Races are not things to be discovered in nature, but they are falsely presented as if they were (Gannett 2004, p. 323).^1^ In turn, version (iii) states that races exist biologically, while noticing that this existence depends on social factors (Outlaw 1992; Kitcher 1999; Spencer 2015). These social factors are intertwined with biological factors, producing real biological divisions. Some philosophers characterize races as bio-social entities, while others discuss how social factors can contribute to establishing biological barriers among racial groups (Outlaw 1992; Kitcher 1999; Spencer 2015). For example, racism can prevent interracial mixing and, thus, it can causally contribute to biological differentiation along racial lines (Kitcher 1999, p. 106).

Finally, in version (iv), “social constructionism about race” refers to the view that race and racial groups exist as social entities. This version of SCR proposes a definition of race and racial groups in terms of social properties or relationships. For instance, races can be groups whose members stand in relationships of power and dominance toward members of other races (Haslanger 2008, 2012). The social nature of races is taken to be analogous to other social entities, such as gender and class (Mallon 2007; Haslanger 2000; Mills 1998). Frequently, this version of SCR is presented as the idea that races are social or human kinds (Sundstrom 2002; Diaz-Leon 2015; Mallon 2016).

Hochman argues that (i) – (iv) are too distinct to form a unified, substantive, and coherent position. (i) is a position about the idea of race, remaining agnostic about the existence of races as things in the world. This view is compatible with (ii), (iii), (iv), or any other metaphysical position about race and racial groups. At the same time, (ii) and (iii) adopt opposing metaphysical positions, denying or accepting that races form biological divisions in nature, respectively. Notice, however, that (ii) and (iii) are agnostic about whether races exist as non-biological entities. Both views are compatible with accepting or rejecting (iv). In contrast, version (iv) claims that there is a sense in which races exist as social entities, but it remains agnostic as to whether races can also exist as biological entities. Hence, one can adopt (iv) and at the same time accept or reject the existence of biological races.

The upshot is that current uses of “social constructionism about race” either refer to or are compatible with widely different metaphysical positions (2022, 5). The constructionist label applies to realists and anti-realists about biological races, as well as realists and anti-realists about races as social categories. In this way, the use of “social constructionism about race” is so ambiguous that it becomes overly inclusive: it is unclear who would be *excluded* from being considered a race constructionist. Philosophers and scientists holding any of the main metaphysical positions about race would fit under the constructionist label. As Hochman nicely puts it, “if social constructionism of race is meant to be an exclusive club, it needs some new bouncers” (2022,7).

Hochman points out two important consequences of his analysis. First, the label “social constructionism about race” is now almost meaningless, having outlived its practical usefulness (2022, 17). After all, this label is not helpful when scholars try to identify and name specific views about race. One cannot use that label to distinguish metaphysical positions and their proponents. For example, it is impossible to use that label to differentiate between those who accept and those who reject the existence of biological races. Second, “social constructionism about race” cannot even fulfil one of its traditional rhetorical roles, namely, expressing an opposition to biological race realism (2022, 8–9). While one has traditionally opposed biological race realism and SCR in the past, current uses of “social constructionism about race” do not imply this opposition. “Social constructionism about race” can refer to four different versions of SCR, but only one of these versions is a rejection of biological race realism. Aside from version (ii), all versions of SCR are either compatible with biological race realism or a type of biological race realism. Thus, the label “social constructionism about race” is so ambiguous that it cannot rule out biological race realists from the “club” of race constructionists.

Hochman’s criticism does not end here.^2^ However, the remainder of this paper will focus on his worries about the ambiguity and over-inclusivity of “social constructionism about race.” I argue that ambiguity is not a serious problem, as that label can still hold practical usefulness. As I elaborate later, that label stands for an investigative practice, i.e., a type of research practice or tradition shared by many race constructionists who might hold different metaphysical views about race.^3^ “Social constructionism about race” is useful because it flags a common research practice among philosophers of race who might hold different metaphysical views. In this sense, I neither challenge Hochman’s four versions of SCR nor the fact that “social constructionism about race” is highly ambiguous.

Issues of ambiguity and over-inclusivity have been raised about the label “social constructionism” in the past. More than 25 years ago, Ian Hacking acknowledged the ambiguity around that label (1999). Hacking offers a broad working definition for social constructionism, but his aim is neither to erase ambiguity nor to make social constructionism an “exclusive club.” Hacking embraces the plurality of views that can fall under that definition (1999, 12–19). One of the key messages in his approach is that constructionist theories are embedded in specific social contexts. These theories develop as historical reactions to certain received views and have practical and normative commitments. Hence, what many social constructionists have in common is not what they specifically say (or metaphysically commit) about a subject but rather *how they investigate it*. In the remainder of this paper, I show that something similar can be said of social constructionism about race.

## Ambiguity and scientific concepts

In recent decades, philosophers of science have given considerable attention to the role of concepts and language in scientific practice (Feest and Steele 2012; Brigandt 2010; 2012; Brigandt and Love 2012; Waters 2014; Neto 2020; Novick and Doolittle 2021; Sterner 2022; Novick 2023). This literature shows that concepts can have different functions besides helping scientists classify phenomena into those that fall and do not fall under the concepts (Brigandt and Love 2012, p. 422). Concepts can be useful even if they are too broad, ambiguous, or imprecise, and do not enable that classification adequately (Brigandt and Love 2012; Waters 2014; Neto 2020). Below, I illustrate this point. This discussion will set the stage for reconsidering social constructionism about race.

Philosophers are often puzzled by imprecision and pluralism in scientific concepts. Why is it the case that many (key) concepts in science either remain so imprecisely or broadly defined that they allow for so many contradictory definitions and uses? This question motivates philosophers to investigate how concepts and their linguistic expressions influence science in practical, social, and unexpected ways. For example, many geneticists work with the concept of molecular gene (Brigandt 2010; Waters 2014). These geneticists study various aspects and phases of protein-coding, such as translation and transcription, using terms like “molecular genes” in very different ways. This term acquires specific meanings in particular contexts and can refer to different parts of the DNA or types of information. At the same time, that term can still be used to convey a very broad notion of molecular gene, something like “structural units that code for protein” (Brigandt 2012).

The broad concept of molecular gene is useful because it identifies something in common among the different labs working on protein-coding, namely a *common explanatory practice* (Brigandt 2010, p. 25). This practice has two components. First, scientists talking about molecular genes have the common explanatory goal of understanding how DNA segments code for various genetic products. Second, those scientists tend to revise their views, definitions, and methods in light of others who share the same explanatory goal. Discoveries from some labs will influence how others characterize molecular genes, and these discoveries can even influence how geneticists will define and redefine expressions like “molecular gene.” Put it simply, scientists working on protein-coding molecular genes form a research community with shared goals and influence (Neto 2020). In this sense, linguistic expressions like “molecular gene” function as signals, or tags, that help scientists recognize when and how they should react to each other’s work.

Another example is the concept of evolutionary novelty (Brigandt and Love 2012; Neto 2020). This concept broadly refers to novel traits in evolutionary history, but scholars widely disagree on what “novel traits” mean (Brigandt and Love 2012, p. 421). The consequence is that the term “evolutionary novelty” is used in different, inconsistent ways across the scientific literature. Another consequence is that there is no single, agreed-upon answer to the question of what counts as an evolutionary novelty. Nevertheless, this concept is still useful because it identifies a *problem agenda*, i.e., an interdisciplinary research topic. This topic organizes scientific practice, bringing together scientists from different fields to contribute in their own way to that topic. More specifically, a problem agenda involves different, heterogeneous, but interrelated questions. These questions must add to the understanding of the general topic and be part of a single, larger historical debate (Brigandt and Love 2012, 422–423).

The concept of evolutionary novelty sets a problem agenda (Brigandt and Love 2012). Scientists working on evolutionary novelty are largely focused on understanding the origin of developmental mechanisms from several different perspectives, such as developmental, genetic, ecological, and phylogenetic. These investigations are interrelated, exploring complementary questions about traits and their distribution across taxonomic groups. Furthermore, the discussion of evolutionary novelty focusing on developmental mechanisms is a historical reaction to what evolutionary novelty meant in a Neo-Darwinian context (Neto 2020, p. 7). In this historical context, developmental considerations were frequently omitted from discussions about evolutionary origin of traits. In contrast, the concept of evolutionary novelty demarcates a new focus and sets of questions. This concept identifies an interdisciplinary area of research, tracking its historical development, and diversity of research questions. In this sense, the term “evolutionary novelty” is the linguistic expression of a problem agenda. It functions as an explicit signal of when and how scientists from different areas are/should engage in the same problem agenda (Neto 2020).

The examples of “molecular gene” and “evolutionary novelty” are suggestive. These terms refer to broad, imprecise, or ambiguous concepts in science. However, precisely because these terms can be used in different ways, they can reflect and allude to social practices that bring scientists together. Scientists engage in common explanatory practices, problem agenda, and other social interactions (Neto 2020). These interactions are often mediated by broad concepts and linguistic expressions, regardless of whether scientists themselves recognize this important mediation role. Furthermore, it is easy to miss this role if one focuses on issues of ambiguity and over-inclusivity. At first sight, if one surveys the uses of “molecular gene” and “evolutionary novelty,” one will likely conclude that these terms are very ambiguous and over-inclusive. However, upon closer look, one will recognize that those terms have not outlived their usefulness. In the next section, I show that an analogous reasoning applies to the expression “social constructionism about race.”

## Social construction as investigative practice

Hochman is correct when observing the ambiguity of “social constructionism about race.” Philosophers and scientists use this expression to indicate different, even contradictory, positions. However, several of these philosophers and scientists share goals, commitments, and assumptions. They approach and investigate the topic of race in a similar vein, i.e., they take part in the same investigative practice. For this reason, as I will argue below, the ambiguous label “social constructionism about race” can function as a tag, identifier, or a signpost of that practice. The label can help us to recognize a social practice shared by scholars who hold different metaphysical commitments about race. I start with a closer look at the notion of investigative practice.

Investigative practices are research programs, traditions, or projects, broadly construed.^4^ Each investigative practice has an overarching research goal shared among scholars. This situation is like the case of “molecular gene,” as molecular biologists share a common, overarching explanatory goal about protein-coding (Brigandt 2010; 2012). However, the goal of an investigative practice does not have to be only and strictly explanatory. For instance, investigative practices can also involve normative commitments. These are moral or practical aspirations or evaluations that will come alongside the epistemic goals of research. Scholars might share theoretical and methodological assumptions as well. Furthermore, these elements of an investigative practice evolve as part of historical debates. There is a historical continuity, such that an investigative practice is established and evolves as a reaction to theories, ideas, or other practices that have been taken for granted until then. This historical aspect was also present in the case of “evolutionary novelty,” a term that tracks the historical development of debates inherited from the Modern Synthesis (Sect. 3). Finally, investigative practices do not have sharp boundaries. It is a matter of degree whether scholars partake in the same investigative practices.^5^

Many self-declared race constructionists in philosophy share an investigative practice (Mills 1998; Haslanger 2000, 2012; Taylor 2013; Jeffers 2013; 2019; Diaz Leon 2015; Mallon 2016; Asta 2018; Msimang 2019). The overarching goal of this practice is to examine *how racialization is a contingent*,* interactive*,* social-historical process with social-political effects*. Those philosophers investigate how humans become racialized through cultural, political, and social factors within certain historical contexts (Blum 2002; Hochman 2019).^6^ In other words, these factors create social perceptions, behaviors, and norms that sort individuals into those groups. While racialization is not entirely independent from biological patterns (e.g., reproduction and inheritance), race constructionists emphasize how these patterns are themselves influenced by cultural, political, and social factors. For instance, anti-miscegenation in the United States contributed to preventing inter-racial marriages, reinforcing the social separation of racialized groups (Oh 2005). Hence, race constructionists explain racialization by focusing on the influence of social relationships, structures, and institutions over individuals (Haslanger 2000; Jeffers 2013; Dias Leon 2015; Asta 2018). Often, that explanation appeals to laws (e.g., Jim Crow), ideologies (e.g., white supremacy), power relationships (e.g., racial inequality), and historical events (e.g., slavery). For this reason, those constructionists are very sensitive to sociological and historical analyses of race relations and racism. They tend to cite, use, and dialogue with social scientists and historians of race and racism.

Self-declared race constructionists in philosophy emphasize the contingency and interactivity of racialization (Mills 1998; Haslanger 2000, 2012; Taylor 2013; Jeffers 2013; 2019; Diaz Leon 2015; Mallon 2016; Asta 2018; Msimang 2019). First, contingency is an important feature of constructionist analyses in general, and it is strongly present among race constructionists (Hacking 1999). Different social factors and historical contexts produce different forms of racialization, none of which is inevitable, fixed, or predetermined. Thus, the effects of racialization (e.g., racism) are neither inevitable nor entirely out of human control. Second, racialization is a dynamic, interactive process. Racialized individuals can themselves reinforce and/or partially resist the social factors shaping racialization (Bowker and Star 1999; Hacking 2007; Haslanger 2019). Hence, the constructionist investigation of racialization tends to highlight how humans react to racializing factors and how these factors (e.g., laws, ideologies, power structures) might adapt in response to those reactions. Humans are not simply portrayed as passive subjects of classification.

The investigative practice shared by many self-declared race constructionists in philosophy also includes normative commitments (Mills 1998; Haslanger 2000, 2012; Jeffers 2013, 2019; Diaz Leon 2015; Mallon 2016; Asta 2018). These commitments are normative aspirations, evaluations, or stances that accompany the claims made by constructionists (Kalewold 2024). These commitments can be more or less explicit and also vary in kind. For example, some constructionists have political purposes that directly and explicitly inform how they define concepts such as race and racialization (Haslanger 2000, 2008, 2012; Mallon 2006). These definitions are supposed to serve as tools for social justice. The work of these race constructionists echoes what Hacking calls “reformist constructionism” (1999, 20). This is the idea that one should adjust the concept of race (or any other socially constructed entity) to reduce the harm that it has caused in history. Lisa Gannett’s work illustrates another type of normative commitment, namely “historical constructionism” (Hacking 1999, 19–21). As I will detail in the next section, Gannett outlines racial assumptions and historical processes that shape genomics science today, which in turn can influence racialization (2004; 2010; 2014). She discusses how science and scientifically-minded philosophers risk reifying the concept of biological race by referring to human genetic “populations” (2004, 340). Gannett’s commitment is to reveal how contingent historical processes led to this notion of population and the mistaken assumption that such populations are objective (1999, 19–21).^7^

The investigative practice of race constructionists is part of a historical debate. I cannot offer a detailed explanation of this history here, but a few comments will suffice to support this claim. Hochman (2022) correctly points out that most race constructionists understand SCR as an alternative to biological race realism. Hochman claims that these two views are not logically incompatible. However, it is important to recognize that SCR is a *historical reaction* to biological race realism and the assumption that the reality of races is a question primarily for the natural sciences. Philosophers of race recognize that SCR is (or was) an alternative to biological race realism because SCR appeared historically as a direct reaction to the hegemony of biology in matters of race. I briefly address this historical point below.

A version of biological race realism was considered prominent until the mid-20th Century (Taylor 2013, Hardimon 2017). This is the racialist race concept, according to which racial groups differ in meaningful ways (e.g., moral and intellectual traits), and this difference is determined by heritable and immutable biological components intrinsic to individuals of each group. This view is often associated with an old-fashioned notion of “natural kinds”, suggesting that those heritable and immutable components form the “biological essences” of each racial group. The upshot is that racial groups have clear-cut, unambiguous boundaries (Bird and Tobin 2024; Gannett 2004).

Historically, the racialist race concept and its old-fashioned (essentialist) view of natural kinds played major roles in legitimizing pernicious social practices like colonialism (Hardimon 2017). Criticisms of these views predate WWII and even the 20th Century, but racialism was still very much present in the West during the first half of the last Century (Huxley and Haddon 1936; Huxley 1936; Barkan 1991). Furthermore, critics and proponents of alternative accounts of race still behaved according to the assumption that race was crucially a subject of the natural sciences (Huxley 1936; Dobzhansky 1941; Reardon 2005; Brattain 2007; Gannett 2013). This assumption is even shared by early versions of race anti-realism (Appiah 1985, 1996). These versions imply that the natural sciences are the ultimate arbiter of whether races exist in the following sense: declaring that races do not exist follows what the biological sciences have to say about human genetic diversity, phenotypic differences, heritability, reproductive barriers, etc. (Lewontin 1972).

The investigative practice shared by race constructionists has historically developed as a *reaction* to the primary role of biology in discussions about race. More precisely, that investigative practice reacts to the racialist race concept, the alternative versions of biological race realism in the mid-20th Century, and the (sometimes implicit) hegemony of the natural sciences in discussions about the reality of race. This hegemony shaped the debate about race, highlighting questions about heritability, reproduction, genetic variation, and other biological processes. In contrast, race constructionists shift the focus to investigations concerning power relations, social institutions, cultural identities, racism, and other social factors that influence racial groups and have concrete consequences for the members of these racial groups. Thus, race constructionists emphasize the role of social sciences in understanding the reality of race. After all, the social sciences are fundamental to uncovering the social-historical processes of racialization. In this way, the investigative practice shared by many self-declared race constructionists imposes minimal metaphysical and meta-metaphysical commitments on them: (i) deny the existence of racialist races and races as essentialist biological kinds; (ii) reject the assumption that the existence of races is fundamentally a subject of the biological sciences.^8^

Let’s take stock. So far, I have described the main features of an investigative practice shared by several self-declared race constructionists in philosophy. These scholars share an epistemic goal (i.e., investigating racialization). They focus on a range of the same social-historical processes, adopt similar normative commitments, and rely primarily on sociological and historical analyses to investigate race. Hence, those race constructionists approach the topic of race in similar ways. This similarity is *practical*, however: it concerns the goals, strategies, and assumptions that shape research. This is the sense in which one can legitimately say that SCR is an investigative practice. SCR is a type of research tradition or practice shared by many self-declared race constructionists.

This new version of SCR can be added to the list proposed by Hochman (Sect. 2). One should recognize at least five versions of SCR rather than four. My version of SCR is not competing with the other versions. To be clear, I am not arguing that there is one single, best way to characterize SCR. There is no one, single, legitimate way to tell who counts as “race constructionist.” Thus, I am neither arguing that SCR is *only* an investigative practice nor that it is *primarily* an investigative practice. As will become clear in the next section, my version of SCR operates at a different level of analysis and applies to scholars who, in principle, defend any of the other four versions of SCR.^9^ The new version only says that, in an *important* sense, to be a race constructionist is to practice philosophy of race in a certain way. In the remainder of this section, I explain why this version of SCR is important. I also explain how it makes the expression “social constructionism about race” practically useful.

It is important to acknowledge that, among other things, SCR is an investigative practice. The notion of investigative practice is an analytical tool that can help philosophers better understand the context of metaphysical debates. This tool foregrounds motivations, research goals, normative commitments, and the historical context influencing metaphysical views. As the concept of investigative practice draws attention to these types of influence, it prompts interesting philosophical questions and new ways of comparing different perspectives on race. For instance, Mallon (2006; 2022) discusses why the debate between race constructionists, race anti-realists, and biological realists persists despite significant agreement among them. He acknowledges that the remaining disagreement largely hinges on metatheoretical differences involving semantic, pragmatic, epistemic, and ethical choices (2022). For example, some metaphysicians develop a view on race according to specific normative commitments, while others do not (2022, 65). Similarly, scholars have different goals and values shaping how they define race (2022, 67).

The notion of investigative practice can help philosophers identify and understand these types of metatheoretical differences and, thus, deep sources of persistent disagreement and agreement. The reason is that metatheoretical assumptions are often implicit in the practice of philosophers, but the notion of investigative practice aims to make sense of this practice. For example, by characterizing SCR as an investigative practice, I am exposing some of the central metatheoretical assumptions shared by self-declared race constructionists. This characterization brings clarity to what race constructionists do and claim. It makes sense of how the position has been established and evolves. It can also help make sense of the connection between race constructionists in philosophy and social constructionism more generally (Sect. 5). More broadly, my version of SCR invites a more systematic analysis of meta-theoretical assumptions and disagreements among scholars, hopefully moving the metaphysics of race debate forward. In the next section, I will expand on the importance and advantages of my characterization of SCR. Before that, let’s consider how this characterization vindicates the usefulness of “social constructionism about race.”

I started this section by discussing how concepts can be useful in science. The concepts of molecular gene and evolutionary novelty are two examples (Brigandt and Love 2012; Neto 2020). These concepts are useful because they identify types of social practices in science (common explanatory practices and problem agenda, respectively). Then, I have shown that many self-declared race constructionists engage in *another type* of social practice, namely, an investigative practice. This practice is shared by constructionists holding different metaphysical views. For example, according to Hochman’s analysis, Haslanger (2000) and Gannett (2004) defend different versions of SCR. Both still share the same investigative practice, however. This suggests that the expression “social constructionism about race” can still be useful by identifying, signalling, or suggesting that scholars with different views about race still share the same social practice. That expression is not helpful if one is trying to identify who holds the same views or metaphysical commitments. In other words, if the “practical usefulness” of that expression depends on its capacity to sort out who has similar metaphysical views, the expression might not be very useful after all. However, “social constructionism about race” is helpful when one is trying to understand how race constructionism is *often practiced*. That expression explicitly appears in the work of many self-declared race constructionists who shared the same investigative practice (Mills 1998; Haslanger 2000, 2012; Gannett 2004, 2014; Jeffers 2013, 2019; Diaz Leon 2015; Mallon 2016; Asta 2018). In this way, the expression is often implicitly associated with that practice, such that tracking the occurrences of that expression in the literature helps one understand similarities in research practice despite dissimilarities in metaphysical commitments.

At this point, one can understand why the ambiguity of “social constructionism about race” is not such a big problem. This expression is ambiguous, allowing dissimilar views to count as instances of race constructionism. At the same time, precisely because there are dissimilar views, it becomes even more valuable to identify points of similarity among scholars. The expression “social constructionism about race” is suitable for this identification. After all, the expression is already suggestive that such a similarity might exist. Furthermore, this expression already signals this similarity, not in virtue of having an explicit or textual meaning (e.g., Hochman’s (i) to (iv)), but in virtue of being actually and often *used* in the context of similar research practices.^10^ Analogously, terms like “molecular gene” and “evolutionary novelty” are important because they are used in the context of similar practices regardless of the explicit or textual meaning these terms display (Sect. 3). In summary, ambiguity does not entail a lack of practical usefulness. This point goes for scientific terminology but also “social constructionism about race.”

## Objections and implications

The view of SCR as investigative practice requires careful elaboration. While it would not be possible to offer an exhaustive analysis of that investigative practice in this paper, I will further flesh out this idea by addressing some objections and implications of my view. This discussion will also provide further evidence as to why “social constructionism about race” is practically useful.

One might object that my view does not adequately avoid Hochman’s original concerns about ambiguity but rather amplifies them. As I explained earlier, Hochman shows that the expression “social constructionism about race” refers ambiguously to four versions of race constructionism. These versions are so different in their metaphysical commitments that “social constructionism about race” is too ambiguous. My proposal makes this problem even worse because it adds another version of race constructionism to the list.

This objection misses the point of my proposal. On the one hand, I have indeed identified a fifth version of SCR (i.e., social constructionism about race is an investigative practice), increasing the ambiguity around “social constructionism about race.” On the other hand, my proposal is not an attempt to eliminate or even reduce that ambiguity. Instead, I argue that ambiguity does not entail a lack of practical usefulness. Sometimes, ambiguous labels are useful precisely because they suggest similarities among scholars with different views. The expression “social constructionism about race” is like that. Besides all its other uses listed by Hochman, the expression can signal a way of investigating the topic of race shared by several philosophers of race. The expression draws our attention away from the potential differences among many self-declared race constructionists, inviting readers to think about the similarity among them. However, this similarity is of a practical, non-metaphysical sort. The ambiguous expression “social constructionism about race” is useful because it is suggestive of that similarity. This usefulness will become clearer later in this section after I consider other objections and implications.

Another objection concerns over-inclusivity. One might argue that too many scholars would count as race constructionists if one understands social constructionism about race as an investigative practice. This notion might be incapable of distinguishing SCR from biological race realism and even race antirealism. For example, biological race realists and antirealists also talk about social-historical processes that influence racialization (Spencer 2015; Hochman 2017). These scholars acknowledge and might even discuss factors such as colonialism, slavery, segregation, power relations, etc. Moreover, race antirealists sometimes also adopt clear normative commitments, taking upon the task of “unmasking” false and pernicious beliefs on biological races (Appiah 1996; Hochman 2017, 2021a). Biological realists might also adopt some of those commitments at least implicitly, for instance, if they think that addressing racial health disparities requires the belief in biological races. For these reasons, my account of race constructionism might be too inclusive. This is a serious charge and deserves careful analysis.^11^

The work of recent proponents of biological race realism is significantly distinct from the investigative practice I have been discussing. Consider the work of Spencer (2012; 2015; 2019). As Hochman points out, Spencer acknowledges that social factors causally influence biological racial divisions (Spencer 2015). These divisions might involve biological facts about genetic differentiation, but these facts are themselves influenced by how individuals behave socially (e.g., what ethnic relationships they establish can influence patterns of reproduction and gene distribution). Thus, biological races only exist as results of social processes. Furthermore, Spencer’s argument for biological race realism fundamentally depends on assumptions about how people use language, how some institutions are organized (e.g., how the US OMB organizes the Census), etc. (Hochman 2014). Thus, Spencer might be characterized as a race constructionist after all (Hochman 2022). Spencer himself rejects the idea that his self-proclaimed biological race realism stands in opposition to SCR (2015).

The view of SCR as an investigative practice helps us understand an important sense in which Spencer engages in a different type of research project than many race constructionists. A crucial aspect of his metaphysical project is a theory of “real biological entities”, namely his appeal to epistemically successful practices in population genetics and other areas of biology (2015; 2016; 2018; 2019). This theory informs *how* Spencer investigates racial divisions and the social factors that influence them. While these factors are acknowledged, he does not engage substantially with norms, laws, ideologies, power relations, and other social aspects of racialization typically discussed by race constructionists. For Spencer, social factors matter in two fundamental ways: (i) they help explain the reference of racial terms; (ii) they are causal influences on biological divisions that are explored by population geneticists (2019).^12^ However, in practice, Spencer’s metaphysical analysis focuses mainly on examining the epistemic practices of population geneticists, for example, by discussing how population geneticists use cluster algorithms, what types of generalizations they produce, etc. This examination takes precedence over explaining the complex relationship between social and biological factors. In other words, even assuming that social factors influence the biological reality of races, the explanatory focus is on how science works rather than how social factors contribute to biological differences that are picked up by successful science. Furthermore, Spencer’s theory does not explicitly involve normative commitments of the type shared by race constructionists. Spencer is not trying to explicitly “unmask” false views about race, or “reform” the concept for practical use. He might be implicitly or personally motivated by some normative goal or another, but this is not a standard that necessarily and explicitly determines the *success* of his theory. It is also hard to characterize Spencer’s theory as part of a historical reaction to bio-centric approaches to race. For these reasons, Spencer is not part of SCR as an investigative practice.

Frequently, self-proclaimed biological race realists are pluralists (Andreasen 2004; Hardimon 2017; Spencer 2018). They argue that races can exist as biological entities in some contexts and as socially constructed entities in others. The notion of investigative practice adds nuance to pluralism. Some pluralists partake in the investigative practice of race constructionists, while others do not. One paradigmatic example of the former is Hardimon (2017). According to him, “Socialrace” picks out real social kinds explicitly understood as socially constructed entities (2017, 131). The reality of these entities results from social hierarchies, systems of oppression, norms, etc. At the same time, Hardimon talks about “minimalist race” and “populationist race,” two notions of race that correspond to biologically real entities. These entities are influenced by social factors, but Hardimon insists that they are biologically rather than socially real (2017, 125). In this sense, Hardimon is a pluralist when it comes to metaphysical claims and commitments. Socialraces are socially real, while minimalist and populationist races are biologically real.

Does Hardimon partake in the investigative practice of race constructionists? Yes, at least when/where he examines SocialRaces. Hardimon’s discussion of “Socialraces” clearly exhibits most features of the constructionist investigative practice. Hardimon engages substantially with the social aspects of racialization, adopts the commitment of “unmasking” false views about race, and emphasizes the emancipatory character of that race concept (2017, 131–132). He articulates the relationship between “Socialrace,” racism, and institutions that perpetuate racism. In this portion of his work, Hardimon engages in the same practice as Haslanger, Jeffers, and other race constructionists within but also beyond philosophy (Sect. 4). This portion of Hardimon’s work is strikingly different from his other discussions as it moves beyond a bio-centric approach to race. His partial adherence to the investigative practice of SCR contrasts with the work of other race pluralists. For instance, Spencer calls himself a “radical pluralist,” but this pluralism is only restricted to metaphysical claims and commitments. As I have explained above, Spencer does not share the investigative practice of those race constructionists.^13^

Another interesting case of a self-proclaimed biological race realist is Andreasen (2000, 2005). Andreasen is known for defending a cladistic notion of race, according to which human races are monophyletic groups (i.e., formed by a common ancestor and all and only its descendants). Andreasen argues that this notion of race corresponds to biologically real entities, but it is also compatible with the existence of socially constructed races (2000, S664). This recognition does not entail that Andreasen shares the investigative practice of race constructionists, however. Andreasen acknowledges important differences in practice between what she does and what she calls the “constructivist project” (2000, S662-663). Constructionists explore the role of race in social practices and racism, typically aiming to expose “myths” to move society beyond racism. In contrast, Andreasen’s project concerns whether a biologically (monophyletic) defined notion of race is scientifically legitimate. This clear difference indicates that, in an important sense, Andreasen is not a race constructionist.

These considerations suggest that the investigative practice of race constructionists is not over-inclusive. This investigative practice excludes prominent biological race realists while also acknowledging the nuances of pluralism. These considerations invite us to reconsider the supposed clash between SCR and biological race realism. As I explained previously, Hochman rejects this clash by arguing that those views are not logically incompatible. I agree with Hochman on this point. If we treat SCR and biological race realism as views about race, they might not contradict one another. However, my analysis introduces another level of analysis, i.e., a new way of comparing those positions. If we characterize SCR as an investigative practice, we should ask whether this practice differs from how biological race realists carry out their research.

The difference in practice is considerable and has historical roots. The investigative practice of SCR evolves as a historical reaction to biological race realism and its bio-centric approach to race in the mid-20th Century. This history has influenced the goals, assumptions, and commitments of many self-declared race constructionists (e.g., Mills, Taylor, Haslanger, Jeffers, Gannett, etc.), contributing to a chasm between how these scholars investigate race and how biological race realists carry out their research today (e.g., Andreasen, Sesardic, Spencer, and partly Hardimon). Biological realists focus on the explanatory success of population categories in the biological sciences (Andreasen 2004; Hardimon 2017; Spencer 2019). They concentrate on how science uses those categories to explain genetic variation, adaptations, evolutionary history, and genetically based diseases. For them, claims about the reality of races are warranted by examining the epistemic practices and successes of the natural sciences. While those claims might also rely on acknowledging social processes (e.g., how slavery might have shaped reproductive patterns), the focus of philosophical analysis is mostly science and its results. Almost no attention is given to how science itself is embedded in society, subject to human biases, and contingent social factors. No attention is given to the relationship between the explanatory practices of science and issues of racism or racial identity. In contrast to the investigative practice of SCR, biological race realists are less explicitly driven by normative commitments.

At this point, it is important to emphasize that investigative practices have no strictly sharp boundaries. Some biological race realists might be closer to the investigative practice of SCR than others, as exemplified by Hardimon’s pluralist approach. This does not mean that there is no significant difference between the investigative practice of SCR and biological race realism. Instead, it means that investigative practices have vague boundaries and, thus, the clash is a matter of degree.^14^ As far as SCR clashes with biological race realism, this clash is more apparent if one considers how scholars investigate race rather than their metaphysical commitments and claims.

My analysis also offers a new way of comparing SCR and race anti-realism. As explained in Sect. 2, Hochman notices an overlap between these two views, showing that “social constructionism about race” is sometimes used to express the view that biological races do not exist (2022). He illustrates this point with the work of Gannett (2004). This work defends race constructionism, but its metaphysical commitments seem to be more aptly described as anti-realist (Hochman 2022, p. 4). While Hochman might even be correct in this assessment of metaphysical commitments, my analysis of investigative practices offers a more complete characterization of her research project. It enables us to understand why Lisa Gannett’s overall research is a prime example of race constructionism, as I show below.

Lisa Gannett’s work does not simply or even mainly reject the existence of biological races. Her focus is on showing how the quasi-racial populations (e.g., continental groups) studied in human genomics are laboratory constructions resulting from historically contingent methodological decisions (2004; 2010; 2014). Her work focuses on the contingencies of genomics science as a way of challenging the tendency to reify or naturalize those constructed populations. In this sense, her work is an explicit attempt to “unmask” this tendency. This work challenges the reasoning of many biological race realists, who extrapolate the biological reality of races from those constructed populations (Kitcher 1999; Andreasen 2000; Sesardic 2010). Furthermore, Gannett describes how those constructed populations can influence and be influenced by racial assumptions with racist effects (2004; 2010; 2014). Her work is not about how science works and how it disproves race; it concerns how science influences and is influenced by social views of race, racial identity, and racialization. In this sense, Gannett’s work exhibits the goals and normative commitments associated with other race constructionists within and outside philosophy (Hacking 1999, 2007; Fullwiley 2008; Bolnick 2008). Her work reacts to the resurfacing of biological race realism in the aftermath of the Human Genome Project, heavily interacting with constructionism in the social sciences. Like several constructionists in this field, Gannett reacts to the emergence of population genomics as a “new science of race”, i.e., to the hegemony of natural sciences as in the discussion of race (Duster 2005; M’Charek 2005; Bolnick 2008; Fullwiley 2008; Bliss 2012; Tallbear 2013). For these reasons, Gannett is a paradigmatic example of someone whose research is driven by SCR as an investigative practice.

To describe Gannett as an anti-realist is to miss all that. This description overlooks her goals, normative commitments, theoretical background, and the type of academic community Gannett interacts with. In turn, my analysis draws attention to these elements and allows for a different comparison between SCR and race anti-realism: besides asking whether Gannett and race anti-realists share the same metaphysical commitments, one can now ask to what extent they share the same investigative practice. This comparison will likely generate interesting results. For instance, the work of early race anti-realists was still committed to a bio-centric approach to race, assuming a biological notion of race to reject the existence of biological races (Appiah 1985, 1996; Zack 1993). The investigative practice of race constructionists is partly a reaction to this approach and, thus, quite different from race anti-realism. Those early anti-realists are excluded from that investigative practice. However, recent proponents of race anti-realism increasingly incorporate theories about racialization as the formation of social “racialized groups” (Glasgow 2008; Hochman 2017). The more scholars develop these theories, the closer they tend to get to SCR as an investigative practice. Indeed, the explanatory goals of those theories are very similar to what race constructionists aim to offer. Hence, another benefit of my analysis is to make sense of how race anti-realism has changed in recent decades and how it looks increasingly like race constructionism.

It is worth considering one last point of comparison between race anti-realism and SCR. As an investigative practice, SCR can unveil how racialized groups came into being and how social influences shape beliefs about race. On the one hand, Hochman and other recent proponents of race anti-realism might want to rely on this investigation to strengthen their arguments. The investigation of social constructionists might be instrumental in advancing anti-realist arguments in the metaphysics of race. Thus, race anti-realists might find it useful to engage even more closely with SCR. On the other hand, the investigative practice of social constructionists is compatible with recent versions of race anti-realism, but it neither entails nor is entailed by these versions.^15^ This is partly due to a series of semantic assumptions made by race anti-realists (Hochman 2016, 2017, 2019). The practice of SCR does not necessitate those assumptions.

Now let’s turn to expressions like “social constructionism about race.” Is this expression almost meaningless? Not necessarily. Hochman thinks that “social constructionism about race” is almost meaningless because scholars use it textually when referring to very different, even contradictory views. In particular, the same expression applies to scholars committed to either realist or anti-realist views about biological and/or social race (Sect. 2). Thus, the expression is so ambiguous in its meaning that it becomes over-inclusive and loses practical usefulness. My problem with this analysis is its reductive understanding of practical usefulness. As I have shown in Sect. 4, linguistic expressions might be useful because they are used by scholars engaged in the same social practice, such that those expressions can signal or track the practice. However, it is worth addressing a final objection related to this point.

One might argue that my suggestion about the practical usefulness of “social constructionism about race” misses important aspects of that expression.^16^ “Social constructionism about race” is currently and explicitly used by philosophers of race to denote a metaphysical position (or set of metaphysical positions). Thus, it seems odd to argue that such an expression is referring to an investigative practice instead. My suggestion also does not help to make sense of the fact that, very often, philosophers claim that race itself is a social construct. While these are legitimate concerns, they miss the scope of my proposal. I do not argue that “social constructionist about race” refers *explicitly* to an investigative practice rather than a set of metaphysical positions. I am not proposing a new meaning for that expression, one that should be adopted at the expense of any other. The point of my proposal is to show that, alongside explicit textual meanings, expressions can have practical functions. One of these functions is to tag, signal, or label the work of scholars who partake in a shared investigative practice.

## Conclusion

Social constructionism about race (SCR) is usually described as a metaphysical view about the existence of race and racial groups. In this paper, I have shown that SCR is also an *investigative practice*, i.e., a historically evolving research program or tradition shared by many philosophers. These scholars share research goals, assumptions, and normative commitments as they aim to understand the social-historical processes that influence racialization. While race constructionists today might have different metaphysical views on race, many of them participate in the same investigative practice. Historically, this practice evolved as a rejection of biological race realism and the assumption (also shared by early race anti-realists) that gives the natural sciences the privilege to examine the reality of race. By proposing this version of SCR, I am shifting attention away from what race constructionists claim towards what they actually do.

My proposal engages critically with the work of Adam Hochman. Hochman has been responsible for revitalizing race anti-realism, as well as extensively criticizing both biological race realism and SCR (2013, 2014, 2017, 2019, 2021, 2022, 2024). Recently, Hochman argued that the expression “social constructionism about race” has outlived its usefulness (2022). My proposal indicates why Hochman is wrong. While Hochman correctly points out the ambiguity of this expression, one should recognize that ambiguity does not entail a lack of practical usefulness. I have shown that “social constructionism about race” is a useful label for the investigative practice shared by many self-declared race constructionists. Finally, this practice is radically different from how several prominent biological race realists conduct research. In this sense, SCR is still an alternative to biological realism.

The notion of investigative practice needs further exploration. This notion is an analytical tool that might prove useful in understanding how metaphysical views about race are embedded in larger socio-historical contexts. It helps us to identify and foreground the goals, motivations, commitments, and other metatheoretical assumptions that influence the metaphysics of race work as philosophers and metaphysicians of race. Hence, the notion of investigative practice can help us, philosophers of race, to be even more reflexive about our own work. This reflexivity is an important step towards recognizing the source of meaningful disagreement among philosophers of race (Ludwig 2015, 2016; Mallon 2006, 2022; Neto 2025).

## Footnotes

1 Hochman presents textual evidence of philosophers using the expression “social constructionism about race” as a rejection of biological race, i.e., as race anti-realism. I will not dispute this evidence here, but I will re-examine the distinction between race constructionism and anti-realism in Sect. 5.

2 Hochman criticizes three out of the four versions of SCR. He argues that version (i) is too broad, and versions (iii) and (iv) are false. The only correct version is (iii), but this position would be more precisely characterized as “race anti-realism” rather than social constructionism about race. These claims face criticisms. For example, Msimang (2019) describes several strategies that one could use to attempt to block Hochman’s criticism of version (iv) (2019, p. 13 and footnote 7). I do not take a stance on this debate here.

3 Here I focus on a research tradition within the philosophical community. There is a case to be made about the connection between race constructionism in philosophy, social sciences, and constructionism more broadly (Hacking 1999).

4 The concept of investigative practice can be defined in different ways (Feest and Steele 2012). My definition is meant to make sense of research around the topic of race rather than to offer a general definition of research practices. Similar concepts are widespread in the philosophy of science literature, such as the notion of “systems of practice” (Chang 2014). These concepts can be more or less vague and narrow in scope, but they all draw attention to the fact that the production of knowledge is embedded in social communities that share goals, commitments, norms, etc.

5 For instance, not all scholars in the same investigative practice must share all assumptions and normative commitments characteristic of that practice. We can understand investigative practices as *clusters*, and scholars instantiate these clusters insofar they share some assumptions and commitments. I return to this point in Sect. 5.

6 Scholars debate whether racialization produces races versus racialized groups, and whether the term “racialization” is better understood as a purely descriptive (pattern) or causal concept (Hochman 2019; Uyan 2021). I do not engage with these debates here. For the purposes of this paper, my goal is solely to identify what I take to be the main research goal of many self-declared race constructionists, namely, to explore the social causes and effects of practices that divide humans into what are traditionally perceived as “racial” groups.

7 The former is committed to showing how an entity is not inevitable but rather a result of historical, contingent, and social-institutional processes. The assumption here is that the entity has been taken for granted as an inevitable part of nature, but it should not. The latter is committed to exposing the practical ideological function that certain conceptions of an entity might serve (e.g., the notion of population in human genomics might rely on and perpetuate racial assumptions).

8 Philosophers of the social sciences debate whether one can plausibly infer metaphysical commitments from the social sciences (Lauer 2022; Hawley 2018). For instance, a pragmatic position would suggest that scientific interest and success legitimizes the postulation of certain entities, but this does not entail that such entities are real in an absolute, mind-independent, or outside-of-a-frame-of-reference way (Lauer 2022). This pragmatism aligns with (ii), and it allows for the co-existence of different (and even contradictory) views about race. While there might be affinities between my view of SCR and that pragmatic position, there is no entailment between the two views. For example, race constructionists are committed to (i) when sharing the SCR investigative practice, but this commitment could be either pragmatist or realist. I suspect that race constructionists tend to be realist about (i), but this is not entailed by SCR. At the same time, engaging with these and other issues in the philosophy of the social sciences will help to flesh out SCR, as the literature on measuring racial discrimination illustrates (Lauer and Khalifa 2023).

9 As stated by Hochman, the four versions of SCR are very general. SCR (as investigative practice) is compatible with them in part because those versions are so general. For instance, none of the versions specify what is meant by “biological race.” However, SCR (as investigative practice) will clash with the claim that biological races exist if one understands “biological race” in a racialist or essentialist sense. Thus, it is important to notice that SCR (as investigative practice) is not entirely neutral on metaphysics.

10 This distinction might be best characterized as two different levels of analysis. One level focuses on the meaning of terms, while the other focuses on the practical activities and goals that can be achieved when the terms are in use. Notice how my analysis of concepts and language differs from Hochman’s. Hochman focuses on the textual meaning of expressions as a basis for analysis, for example, using explicit textual quotations as evidence of what philosophers mean by “social constructionism about race” (2022, 4–5). In contrast, I notice the broader context in which the expression appears, emphasizing the goals of research. The textual meaning of the expression might vary, but it appears in many texts and circumstances that embody the same research goals and practice. Thus, besides its textual meanings, the expression becomes a tag, or label, associated with a certain way of doing things. This distinction in level of analysis resembles aspects of Brigandt’s theory of concepts (2012).

11 I thank one of the reviewers for pressing this point further.

12 When discussing these social factors, Spencer also does not examine the contingent, historicity, and somewhat arbitrariness of these factors. He also does not examine the social context and influences that surround the scientific practice of population geneticists. These examinations are strongly present in the investigative practice of race constructionists.

13 Thus, one advantage of my analysis is to make sense of how race pluralism can differ among philosophers of race. Spencer and Hardimon are self-declared pluralists, but only Hardimon is a pluralist in how he conducts investigative practices.

14 Analogously, many concepts admit vague boundaries but still capture significant contrasts (e.g., “bald”).

15 I thank one of the reviewers for raising this comparison. The comparison between the investigative practice of race constructionists and race anti-realists deserves closer analysis. I am unable to perform this analysis here without making the paper too long.

16 I thank one of the reviewers for pushing this point.
